# Recall accuracy of weekly automated surveys of health care utilization and infectious disease symptoms among infants over the first year of life

**DOI:** 10.1371/journal.pone.0226623

**Published:** 2019-12-17

**Authors:** Catherine Ley, Lauren Willis, Maria de la Luz Sanchez, Julie Parsonnet

**Affiliations:** 1 Division of Infectious Diseases and Geographic Medicine, Department of Medicine, Stanford School of Medicine, Stanford, CA, United States of America; 2 Division of Epidemiology, Department of Health Research and Policy, Stanford School of Medicine, Stanford, CA, United States of America; Bielefeld University, GERMANY

## Abstract

Automated surveys, by interactive voice response (IVR) or email, are increasingly used for clinical research. Although convenient and inexpensive, they have uncertain validity. We sought to assess the accuracy of longitudinally-collected automated survey responses compared to medical records. Using data collected from a well-characterized, prospective birth cohort over the first year of life, we examined concordance between guardians’ reports of their infants’ health care visits ascertained by weekly automated survey (IVR or email) and those identified by medical chart review. Among 180 survey-visit pairs, concordance was 51%, with no change as number of visits per baby increased. Accuracy of recall was higher by email compared to IVR (61 vs. 43%; adjusted OR = 2.5 95% CI: 1.3–4.8), did not vary by health care encounter type (hospitalization: 50%, ER: 64%, urgent care: 44%, primary care: 52%; p = 0.75), but was higher for fever (77%, adjusted OR = 5.1 95%CI: 1.5–17.7) and respiratory illness (58%, adjusted OR = 2.9 95%CI: 1.5–5.8) than for other diagnoses. For the 75 mothers in these encounters, 69% recalled at least one visit; among 41 mothers with two or more visits, 85% recalled at least one visit. Predictors of accurate reporting by mothers after adjusting for illness in the baby included increased age and increased years of education (age per year, β = 0.05, p = 0.03; education per year, β = 0.08, p = 0.04). Additional strategies beyond use of automated surveys are needed to ascertain accurate health care utilization in longitudinal cohort studies, particularly in healthy populations with little motivation for accurate reporting.

## Introduction

Telephone surveys conducted by interviewers are ubiquitous, collecting information across a vast spectrum of domains—voter turnout, labor statistics, market research, healthcare utilization, and many others. Their pervasiveness is due to their presumed high level of accuracy, at least for objective phenomena [1]. Although interviewer-administered surveys are considered a gold standard for data collection, they are highly resource-intensive. Automated surveys via mobile or web-based technologies—for example, by interactive voice response (IVR, also known as robocalls) or email—have the distinct advantage of being convenient, efficient, and comparatively inexpensive. Automated surveys in medical care are generally well accepted: patient satisfaction, for example, was shown to be increased in patients who received a follow-up call after an ambulatory visit, with no difference between responses to a survey administered by a human caller or by IVR [2]. In some cases, IVR may in fact produce improved response rates compared to interviewer-administered surveys, particularly with respect to sensitive issues (e.g., depression in adolescents [3] or daily alcohol consumption [4, 5]).

Prospective cohort studies depend on the validity of self-report to make accurate and reliable conclusions. Validation of IVR surveys compared to self-administered paper questionnaires or interview by a clinician has been performed for a range of conditions including, among others: weight and height in young adults [6], neuropsychological testing [7]; teen addiction [8]; inflammatory bowel disease [9]; and depression [10–12]. Although several studies have evaluated the accuracy of in-person interviews or self-administered questionnaires to medical records with respect to health care utilization [13–17], pregnancy/birth history [18–22] and medical conditions [23–27], automated survey results have not been similarly compared to medical records either at a single time point or across time [28]. We sought to assess the accuracy of longitudinally-collected automated surveys in comparison to medical records over the first year of life in a multiethnic birth cohort, Stanford’s Outcomes Research in Kids (STORK).

## Materials and methods

### Recruitment, enrollment and follow-up

Detailed methods for the STORK cohort have been described previously [29]. In brief, pregnant women were recruited through attendance at public obstetric clinics or by self-referral. An interested participant was eligible if fluent in English or Spanish and had a mobile phone. Written informed consent was administered at a baseline home visit convenient to the participant; written parental/guardian informed consent for the baby’s participation was obtained once the baby was born. Information collected at the baseline household visit included demographic and household characteristics. Mothers were also asked to provide permission to review their medical records for infectious illnesses during pregnancy and for those in their infant after birth.

A short (less than three minute-long) automated survey (S1 Table) [29] was administered every week after delivery up until age three years regarding the baby’s health. If the mother reported illness in the baby during the prior week, the survey asked about visits to a health care provider (HCP), infectious disease symptoms with their duration, and antibiotic use. If the baby was well, the survey instead asked an identical number of questions as the illness survey but about breastfeeding status, introduction to new foods, and amount of sleep. At the beginning of our study, in 2011, IVR surveys were used; shortly thereafter, however, some participants requested an email version for its greater convenience. Mothers could select or switch between any method (IVR, email or person-to-person) as they preferred, either by contacting the study office or by request at any 4-monthly household visit. Automated telephone calls were placed every 2 hours both Tuesday from 9:30 to 17:30 and Wednesday from 11:30 to 19:30 unless answered; emailed surveys were sent every Tuesday, Wednesday and Thursday at 7:30 unless answered. If no survey response was received after three days, study staff attempted to contact the participant that same week to administer the survey over the telephone.

Compensation for participation included free wash products, a $10 gift card per participant per household visit and a $25 gift card for each 16 weekly surveys completed. The study was approved by Stanford University’s Administrative Panel on Human Subjects in Medical Research and the Santa Clara Valley Medical Center Institutional Review Board (ClinicalTrials.gov identifier: NCT01442701) and supported by the National Institutes of Health [5R01HD063142 and 5R21ES023371].

Chart review from any available medical records was performed to assess diagnoses and any associated prescriptions. Weekly automated surveys were administered using Precision Polling (Palo Alto, CA) if by telephone or Qualtrics (Provo, UT) if by email. Medical record information was abstracted into Medrio (San Francisco, CA) from either electronic medical records or from paper records obtained from HCPs. All data were managed in REDCap [30] hosted at Stanford University.

### Statistical methods

#### Key outcomes

**Fig 1:**
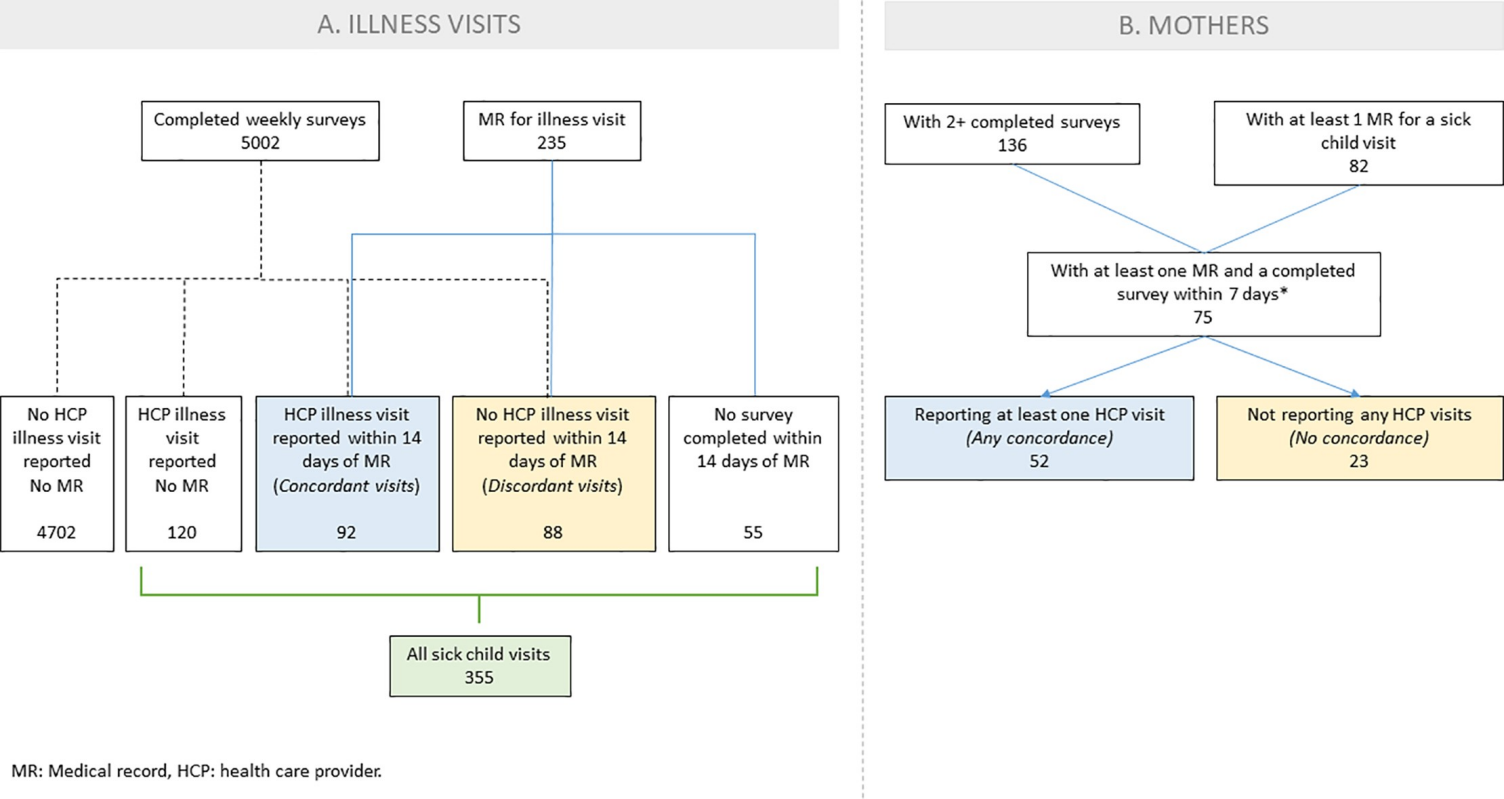
Visit and participant disposition. The analysis set included only visits for illness diagnosed by a HCP and documented in medical records (well-baby check-ups or scheduled visits were not considered); these 235 visits corresponded to a total of 75 babies. Blue boxes indicate: in A, the number of illness visits reported on the available surveys [concordant visits]; in B, the number of mothers reporting at least one illness visit [concordant mothers]. Yellow boxes indicate: in A, the number of illness visits not reported on the available surveys [discordant visits]; in B, the number of mothers who did not report any HCP illness visits [discordant mothers]. The green box indicates the total number of visits for illness either reported (concordant, discordant or neither) or documented (concordant, discordant or neither). HCP: health care provider; MR: medical record.

Because we obtained medical records only from mothers’ and babies’ primary health care facility, and because participants were free to visit unrelated emergency rooms, urgent care and other providers, mothers’ reports of health care visits for illness could not always be confirmed. For this study, then, we chose to evaluate whether recorded illness visits at the primary institution were corroborated by the mother’s survey responses. Recorded illness visits were defined as HCP visits for an illness for which we had medical records (“illness visits”); well-child check-ups or scheduled visits were excluded. We identified all IVR or email surveys that had been completed within 14 days after the recorded HCP visit. If two records were available during this time, only the one most proximate to the illness was reviewed. A record—survey pair was defined as concordant when the first survey response within 14 days of the HCP visit reported that visit. If the first survey within 14 days did not report the visit, the result was considered discordant. If no survey response was received, the visit was excluded from further analysis (Fig 1A). In addition to illness visits, we also chose to evaluate mothers. Mothers who reported at least one HCP illness visit within 14 days after the visit were classified as concordant, while those who did not report any illness visits despite the presence of at least one medical record for illness were classified as discordant (Fig 1B).

Secondary outcomes included the concordance of either a diagnosis of fever or a temperature measurement of 100°F or greater in the medical record and the report of fever in the survey, and the increasing number of concordant visits in mothers across time.

#### Key exposure and co-variables

The primary independent variables were maternal demographic and household factors, including maternal age, years of education and race/ethnicity (White, non-Hispanic; Asian, Hispanic or non-Hispanic; Black, Hispanic or non-Hispanic; White or Other, Hispanic). Co-variables related to study logistics included: the number of days between the first and the last survey completed (survey-days), the number of surveys completed overall, the survey completion rate (surveys completed / weeks in study), the number of surveys where illness was reported (sick-surveys), the proportion of all completed surveys where illness was reported, the total number of medical records and the total number of HCP illness visits (either recorded or reported, or both).

#### Statistics

We tested differences between concordant and discordant visits or between mothers with and without at least one concordant record-survey pair using Wilcoxon rank-sum tests or t-tests for continuous variables and chi-square, Mantel-Haenszel chi-square or Fisher’s exact tests for categorical variables. We assessed the correlation between variables using the Spearman correlation coefficient. We used multivariable logistic regression modeling to explore predictors of concordance among visits and multivariable regression modeling to examine predictors of increasing numbers of concordant visits among mothers.

All analyses were performed in SAS Version 9.4 (Cary, NC).

## Results

A total of 301 HCP illness visits were reported among the 5002 weekly surveys completed over the first year of life for the 136 babies born into the STORK cohort (median number of HCP visits for illness reported: 2; Q1-Q3: 0–3, range 0–13; median number of surveys answered: 42.5, Q1-Q3: 29–46, range: 2–51, with no difference in use of IVR for those who withdrew from the cohort compared to those who did not) (Fig 1, S2 Table). Of these reported HCP illness visits, 120 (40%) could not confirmed by a medical record (median number of such survey reports: 1; Q1-Q3: 1–3, range: 1–10). Separately, a total of 235 illness visits were reported in the medical records of 82 babies (median number of records: 1; Q1-Q3:1–4, range: 1–12). Of these illness visits in the medical records, 55 occurred during a two week period that was not covered by a survey (median number of such records: 1; Q1-Q3:1–2, range:1–8). The remaining 180 medical records, from 75 individual babies, pertained to visits that occurred during a two week period when a survey had been completed, and constituted our analysis sample.

### Visits

**Fig 2:**
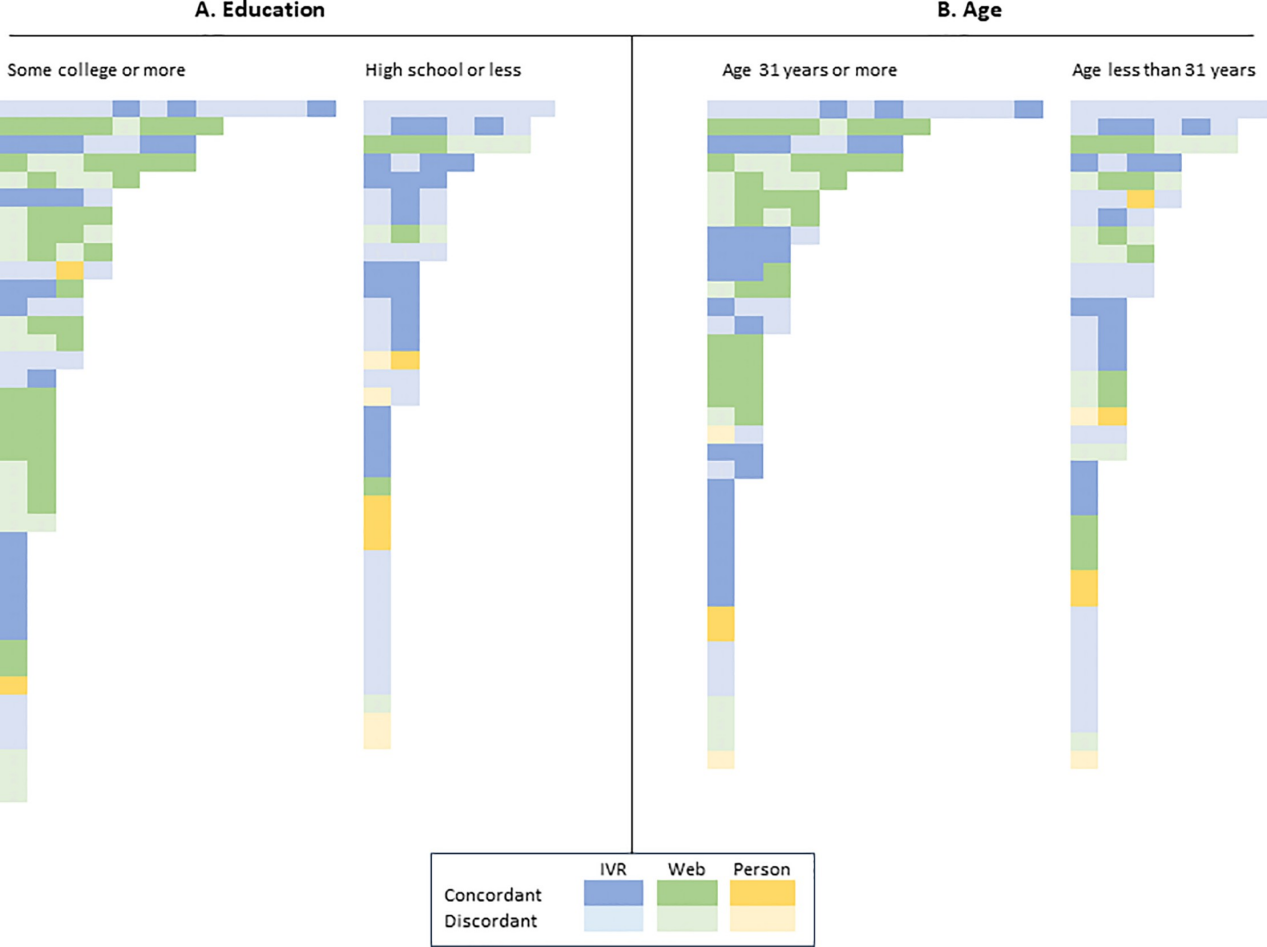
**Distribution of visits per mother, stratified by (A) level of maternal education or (B) age.** Education: any college vs. high school or less; age: less than vs. equal to or greater than the median (31 years). Each square represents one HCP illness visit. Each row within a column represents a mother. Within each column, mothers are sorted by total number of visits (highest to lowest). Visits within each mother are sorted by time, with the earliest visit on the left-hand side of the column. Visits are classified both by type of survey used after the visit (IVR: blue; email: green; person: yellow) and by concordance (concordant: dark; discordant: light).

Mothers in this analysis selected a single method for responding to surveys, with only one woman switching from IVR to email responses over this first year of life. Just over half of surveys were collected via IVR (55%) (Table 1). Accuracy in reporting HCP visits for illness was higher for visits reported by email survey and by person-to-person survey compared to IVR (61% and 60% vs. 43%, p = 0.07). Mothers with least some college, compared to those with a high school education or less, and those aged greater than the median (31 years) compared to those aged less than the median, were more likely to select email surveys, have incurred more visits and have reported them (Fig 2); education was highly correlated with age (r = 0.43, p<0.001). The total number of visits incurred did not affect the concordance rate (p = 0.24) (Table 2).

**Table 1:** Concordance/discordance by diagnosis and location.

	Overall (% of total)	Concordant N (% of row)	P*
	180 (100)	92 (51.1)	
**Data collection method**			
IVR	99 (55.0)	43 (43.4)	0.07
Email	71 (39.4)	43 (60.6)	
Person-to-person call	10 (5.6)	6 (60.0)	
**Location**			
Hospitalizations	4 (2.2)	2 (50.0)	0.75
Emergency room	14 (7.8)	9 (64.3)	
Primary care	119 (66.1)	62 (52.1)	
Urgent care	39 (21.7)	17 (43.6)	
Other^(1)^	4 (2.2)	2 (50)	
**Diagnoses**			
Respiratory infections^(2)^	113 (62.8)	65 (57.5)	0.03
Fever^(3)^	17 (9.4)	13 (76.5)	0.03
Gastroenteritis	16 (8.9)	8 (50.0)	0.93
Conjunctivitis	10 (5.6)	2 (20.0)	0.05
Allergy^(4)^	27 (15.0)	13 (48.2)	0.74
Non-allergic rash	21 (11.7)	10 (47.6)	0.73
Other^(5)^	32 (17.8)	14 (43.8)	0.36

* Fisher’s Exact test

^(1)^ Other location: pediatric gastroenterology, dermatology clinic, telephone call to HCP

^(2)^ Respiratory infections: bronchitis; bronchiolitis; cold; congested/congestion; cough; croup; early viral infection; lower respiratory tract infection; nasopharyngitis; other viral syndrome; otitis media; pharyngeal erythema; possible pertussis; pneumonia; nasal rhinitis; sinusitis; stuffy nose; URI.

^(3)^ Mothers reported the occurrence of a fever for 35 of 46 (76%) visits where the chart indicated either a temperature measurement > = 100°F and/or a diagnosis of fever. Two of these mothers reported the fever but not the HCP visit.

^(4)^Allergy: acute hypersensitivity reaction; allergic rhinitis; amoxicillin rash; atopic dermatitis; eczema; food allergy; neonatal acne; non-specific skin eruption; wheezing/reactive airways disease.

^(5)^ Other diagnoses: anemia, aphtaes ulcers, life-threatening event, aspiration/vitamin E supplementation; blepharitis; bloating; blood stool; breath holding; congenital lacrimal stenosis; constipation; corneal abrasion; cradle cap; diarrhea; dacryocystitis; dacryostenosis; drainage of abscess left buttock; elevated lead; emesis; feeding problems; paronychia; fussiness; tracheomalacia; lymphadenitis; lymphadenopathy; penile irritation; pruritus; post feed emesis; rash; seborrhea; teething; urticaria.

**Table 2:** Agreement across numbers of survey-record pairs.

	Total	Concordant	%
**Visits**			
1	75 (42.7%)	32	0.43
2–3	65 (36.0%)	42	0.65
4–5	22 (12.2%)	9	0.41
6–12	18 (10%)	9	0.50
**Overall**	**180**	**92**	0.51*
**Mothers with:**			
1 visit	34 (45.3%)	17	0.50
2–3 visits	26 (34.7%)	21	0.81
4–5 visits	8 (10.7%)	8	1
6–12 visits	7 (9.3%)	6	0.86
**Overall**	**75**	**52**	0.69**

Test for linear trend

*p = 0.24

**p = 0.02

The majority of visits were to primary or urgent care (66% and 22%, respectively) (Table 1). Concordance by type of health care encounter did not vary across location type, with recall of a hospitalization or an emergency room visit equivalent to that for visits to a primary HCP and other sites, although hospitalizations were few (N = 4) (p = 0.75). Time since birth that the visit occurred was not associated with concordance (p = 0.51).

Of the 180 individual illness visits, 62.8% of all diagnoses (up to three recorded per visit) were related to respiratory infection (e.g., pneumonia, bronchiolitis, otitis media, upper respiratory infection [URI]), with allergic symptoms/diagnoses being the next most common (15.0%) (Table 1). Concordant visits were more likely than discordant visits to include a diagnosis of fever (76.5%, p = 0.03) or one related to respiratory infection (57.5%, p = 0.03) and less likely to include a primary diagnosis of conjunctivitis (20.0, p = 0.05).

Of the 17 diagnoses of fever identified in the medical report, 13 (76%) were subsequently reported by the mother on the weekly survey. Of the 46 instances of either a diagnosis of fever and / or a measured temperature equal to or greater than 100°F as recorded in the medical chart (28 instances had both, five had the diagnosis only), 35 (76.1%) were subsequently reported by the mother (two of these occurrences of fever were reported even though the HCP visit was not).

Logistic regression modeling identified both illness type (fever and diagnoses related to respiratory infections) and survey method as independent predictors of concordance among visits (respiratory infections compared to none: OR = 2.9 95%CI: 1.5–5.8; fever compared to none: OR = 5.1 95%CI: 1.5–17.7; email compared to IVR: OR = 2.5 95% CI: 1.3–4.8).

### Mothers

Based on demographic and household characteristics, the 75 babies and their mothers were similar to the entire STORK sample [29]. Mothers were on average approximately 30 years of age, primarily Hispanic (60.0%), with just over a third having their first child (Table 3). A total of 37% were born outside of the US, 35% spoke Spanish as their preferred language and approximately half had at most a high school education; their homes tended to be crowded, including on average four people of whom at least one was under the age of 18 years.

**Table 3:** Demographic and household characteristics and study logistics, for mothers overall and with and without at least one concordant visit/report pair. Numbers presented are N (%) unless otherwise indicated.

		Overall	Concordant at least once		
		[% of total]	[% of row]		
		(N = 75)	Yes (N = 52)	No (N = 23)	P^(1)^
**Demographic and household characteristics**					
Maternal age (years)	Median (Q1-Q3)	31 (26–34)	31.5 (28.5–34)	28 (23–33)	0.05
Age category (years)	N (%)				0.17^(2)^
<26		17 (22.7)	10 (58.8)	7 (41.2)	
26–30		20 (26.7)	13 (65.0)	7 (35.0)	
31–33		16 (21.3)	12 (75.0)	4 (25.0)	
34+		22 (29.3)	17 (77.3)	5 (22.7)	
Race/ethnicity	N (%)				0.39^(3)^
W, NH		11 (14.7)	10 (90.9)	1 (9.1)	
A, H/NH		14 (18.7)	9 (64.3)	5 (35.7)	
B, H/NH		5 (6.7)	3 (60.0)	2 (40.0)	
W, H or O, H		45 (60.0)	30 (66.7)	15 (33.3)	
First born child	N (%)				0.61
No		49 (65.3)	33 (67.4)	16 (32.7)	
Yes		26 (34.7)	19 (73.1)	7 (26.9)	
Born in the US	N (%)				0.76
No		47 (62.7)	32 (68.1)	15 (31.9)	
Yes		28 (37.3)	20 (71.4)	8 (28.6)	
Preferred language	N (%)				0.99
English		49 (65.0)	34 (69.4)	15 (30.6)	
Spanish		26 (34.7)	18 (69.2)	8 (30.8)	
Years of education	Median (Q1-Q3)	13.0 (11–16)	14.0 (12–17.5)	12.0 (11–14)	0.03
Education category	N (%)				0.02^(2)^
Less than high school		22 (29.3)	12 (54.6)	10 (45.5)	
High school		14 (18.7)\	9 (64.3)	5 (35.7)	
College (any)		22 (29.3)	16 (72.7)	6 (27.3)	
Post graduate		17 (22.7)	15 (88.2)	2 (11.8)	
Minors in household	Median (Q1-Q3)	1 (0–2)	1 (0–2)	1 (0–2)	0.98
Adults in household	Median (Q1-Q3)	3 (2–4)	3 (2–4)	2 (2–4)	0.18
Crowding	Median (Q1-Q3)	1.1 (0.8–1.7)	1.2 (0.8–1.7)	1.0 (0.8–1.5)	0.94
Baby sex	N (%)				0.15
Female		33 (44.0)	20 (60.6)	13 (39.4)	
Male		42 (56.0)	32 (76.2)	10 (23.8)	
**Study logistics**					
Time between first and last surveys (days)	Median (Q1-Q3)	336 (321–344)	338 (325–344)	323 (280–343)	0.03
Surveys answered	Median (Q1-Q3)	43 (34–46)	44 (41.5–47.5)	41 (22–43)	<0.001
Completion rate (surveys/weeks)	Median (Q1-Q3)	0.91 (0.83–0.96)	0.92 (0.88–0.97)	0.84 (0.76–0.93)	0.01
Sick visit surveys	Median (Q1-Q3)	3 (2–4)	3 (2–5)	2 (1–3)	0.005
Proportion of sick surveys (sick surveys / all surveys)	Median (Q1-Q3)	0.07 (0.04–0.12)	0.08 (0.04–0.13)	0.07 (0.04–0.09)	0.46
Medical records	Median (Q1-Q3)	2 (1–4)	3 (2–4)	2 (1–3)	0.04
All sick visits ^(4)^	Median (Q1-Q3)	3 (2–5)	4 (2–6)	2 (1–4)	0.03

^(1)^ Compares mothers with concordant pairs to those without concordant pairs.

^(2)^ Mantel-Haenszel Chi-Square test

^(3)^ Fisher’s Exact test

^(4)^ Any illness visit, including those reported by survey, by medical chart review or both.

Of these 75 mothers, 52 (69%) had at least one concordant visit (median number of visits: 1, Q1-Q3:0–2, range: 0–7) (Table 2). Mothers with at least one concordant visit were slightly older than non-concordant mothers and had more years of education; they were also more likely to be enrolled longer in the cohort, answer more surveys and report more sick visits (Table 3). While white non-Hispanic mothers had a high concordance rate compared to other race/ethnic groups (90.9% vs. 60–67%), this distribution was not statistically significant (p = 0.39). Regression modeling identified both maternal age and maternal education as independent predictors of increasing numbers of concordant visits after adjustment for a measure of illness in the baby (total number of illness visits either reported, recorded or both) (age per year, β = 0.05, p = 0.03; education per year, β = 0.08, p = 0.04).

## Discussion

Our goal in this study was to determine the agreement between any maternal report of an illness visit via automated weekly survey and the actual medical record from the HCP for infants followed over their first year of life. We found that overall only 51% of visits were accurately reported by the mother, with no difference across visit locations or with the total number of visits that occurred. Visits and reports for fever or respiratory infections (including otitis media) were more likely to be concordant than those for conjunctivitis, allergy or non-allergic rash, and reports and surveys by email or with an interviewer were more likely to be concordant than those by IVR. Among mothers, a total of 69% accurately reported that their sick baby had been seen at least once by a clinician; agreement increased to more than 80% among older mothers, among those with higher levels of education, and among those with multiple records from HCP visits.

During their first year of life, more than half of infants in our cohort had three or fewer illness-related HCP visits as per IVR report compared to two or fewer per medical record review. This rate of illness is within the range of other studies in healthy babies [31–34]. The discrepancy between reports and records could have been due to either incomplete medical record ascertainment or over-reporting by mothers; we did not have records for visits at sites not affiliated with the primary HCP. Accuracy in parental recall of the type and frequency of health care service visits has been shown to be quite variable, with recall of hospitalizations generally considerably better than that of emergency department or outpatient visits [35]. In our sample, recall of a visit after one week was accurate in only 50% of four hospitalizations recorded by automated survey in the first year of life; this proportion was not statistically different from that for visits to emergency, urgent or primary care.

Variability in recall is associated in part with both the time frame and the research question of interest [15, 16, 36]. Additionally, whether recall is improved with increased severity of the condition may be highly sample- and methodology- dependent. In our generally healthy cohort, sick child visits for fever or respiratory conditions– 9% and 63% of all visits, respectively—were recalled slightly more accurately than for other conditions, on the order of 77% and 58%, respectively. A study of randomly selected Group Health Cooperative members in Washington state examined the ability of parents to recall injuries in their young children by telephone interview and showed that recall after two weeks ranged from 93% for major injuries to 71% for minor ones; recall one year later ranged from 56% to 19% for major and minor injuries, respectively [37]. On the other hand, in a highly motivated Dutch cohort at high risk of atopic conditions, agreement between the in-person maternal report at a doctor’s interview every six months compared to the medical record was above 90% (range 77–100%) for most childhood illnesses [26].

Prior studies have suggested that recall of health-care related events can be affected by a wide range of demographic factors. We did not find that the number of children in the household affected agreement rates, suggesting that in this cohort, mothers of first-borns were not overly obsessed about reporting their child’s health nor were mothers with many children less likely to report what happened to their youngest. Confirming other studies, we found that education level was strongly associated with accuracy, with mothers with less than a high school education having the lowest agreement rate, and those with post-graduate education the highest. Education level has long been associated with both baseline and longitudinal participation in research studies [38–40]. It is possible that, despite an extensive informed consent process, our cohort participants’ understanding of the purpose of research and what was expected in terms of their participation (e.g., the time commitment needed for an accurate weekly report) was not fully appreciated by those with fewer years of schooling. In support of this, non-concordant mothers completed fewer surveys during their time enrolled compared to concordant mothers (84 vs. 92% of surveys). Finally, maternal age was a predictor of concordance. This finding may simply be due to participation bias from our study’s location in, or affiliation with, an academic research environment, with potential participants perhaps more familiar with research requirements. Alternatively, increased age may be a marker for both increased education and higher income [41], shown previously to be a predictor of agreement [42, 43].

The scientific literature contains very little information on the accuracy of IVR-collected reports compared to medical record review. A pilot study of an IVR tool used in a hospital setting in Ghana showed agreement between childhood illness symptoms reported by the parents and those observed by physicians of 84, 82, 84 and 76% for fever, cough, diarrhea and vomiting, respectively [44]. These findings suggest that in appropriate settings, IVR surveys can be highly accurate. Most IVR interventions to date, however, appear to focus on improving patient follow-up/reminders and medication adherence [45], rather than data collection over time as in our cohort. Our weekly automated survey was short, focusing on illness and any HCP visits associated with illness within the prior seven days and took fewer than three minutes to complete if by IVR. Nevertheless, accurate response rates were far lower than we had anticipated, calling into question the use of IVR methods for longitudinal studies, particularly in healthy populations that are not highly motivated to give accurate responses.

Limitations to our analysis include the fact that the medical records for each baby were not comprehensive, so we could not evaluate standard agreement measures (e.g., kappa statistics). Families within the San Francisco Bay Area have access to a vast number of medical care services and it was not possible to retrieve all visit records. Accordingly, the medical records that we did have were considered our”gold standard,” and we defined concordance in this study as the proportion of medical records where the mother reported a HCP visit within the prior week. It is possible that the request to report only sick-child visits on the automated survey created confusion with well-baby or other regularly scheduled visits, so the mother may have underreported these visits. We limited our analysis, however, to consider only those matched pairs that were available. Finally, a key underlying concern with recall over time is that mothers with sick children may be simply be unable or unwilling to report accurately even if by automated survey, possibly due to the short time frame of interest. It would be important to assess whether the accuracy of a bi-weekly or a monthly survey would be improved over one administered weekly, as these time frames may be preferred [28].

In summary, our study in a multiethnic birth cohort suggests that the use of weekly IVR or email surveys to report childhood illness does not reflect health care visits accurately. Additional effort is needed to generate accurate reporting systems, particularly in healthy populations that might have little motivation for accurate reporting.

## Supporting information

S1 File Data dictionary. Data dictionary for data files S2 File and S3 File.
(XLSX)

S2 File Data per mother. Per mother data including number of concordant visits.
(CSV)

S3 File Data per visit. Per visit data including survey type, location of visit, diagnoses and concordance status comparing the survey report to the medical record.
(CSV)

S1 Table Questions included in the weekly survey, as administered by IVR, email or person-to-person. (DOCX)

S2 Table Survey collection methods and demographic characteristics of mothers in the cohort overall, and in those who withdrew within the first year of the study compared to those who did not. (DOCX)
