# Supplementary material for: Recall accuracy of weekly automated surveys of health care utilization and infectious disease symptoms among infants over the first year of life
Source: PLoS One. 2019 Dec 17;14(12):e0226623. doi: 10.1371/journal.pone.0226623 (PMC6917293; doi:10.1371/journal.pone.0226623)
Supplement: S1 Table — (DOCX) [file pone.0226623.s004.docx]

**S1 Table. Questions included in the weekly survey, as administered by IVR, email or person-to-person.**

| 1 | In the last 24 hours did your baby cry for more than 3 hours straight, unable to be comforted? | Yes/No |
| --- | --- | --- |
| 2 | Has your child used a diaper this past week? | Yes/No |
| 2.1 | If Yes [to Question 2]:  Has your baby had diaper rash in the past 7 days? | Yes/No |
| 3 | In the past week, has your child been sick or unwell at any time? | Yes/No |
| 3.1 | If No [to Question 3]: |  |
| 3.1.1 | Did your baby drink any of the following in the last 24 hours? |  |
| 3.1.1.1 | Breast milk | Yes/No |
| 3.1.1.2 | Formula | Yes/No |
| 3.1.1.3 | Cow milk or other type of milk | Yes/No |
| 3.1.2 | Did you introduce any new foods to your baby in the past 7 days? | Yes/No |
| 3.1.3 | In the last 24 hours, how many hours in a row was the longest stretch of time that your baby slept? | # |
| 3.2 | If Yes [to Question 3]: |  |
| 3.2.1 | How many days in the past 7 days has your child been sick? | # |
| 3.2.2 | Did your child visit a health care provider for any health concern in the past 7 days (not including routine, scheduled appointments)? | Yes/No |
| 3.2.3 | How many days in the past 7 days did your child have a cold or runny nose? | # |
| 3.2.4 | How many days in the past 7 days did your child have a cough? | # |
| 3.2.5 | How many days in the past 7 days did your child have a fever? | # |
| 3.2.6 | How many days in the past 7 days did your child have ear tugging / pulling? | # |
| 3.2.7 | How many days in the past 7 days did your child have diarrhea? | # |
| 3.2.8 | How many days in the past 7 days did your child have vomiting? | # |
| 3.2.9 | Did your child take antibiotics in the past 7 days? | Yes/No |
| 3.2.9.1 | If Yes [to Question 3.2.9.2]:  How many days in the past 7 days did your child take antibiotics? | # |
