# Supplementary material for: Recall accuracy of weekly automated surveys of health care utilization and infectious disease symptoms among infants over the first year of life
Source: PLoS One. 2019 Dec 17;14(12):e0226623. doi: 10.1371/journal.pone.0226623 (PMC6917293; doi:10.1371/journal.pone.0226623)
Supplement: S2 Table — (DOCX) [file pone.0226623.s005.docx]

**S2 Table: Survey collection methods and demographic characteristics of mothers in the cohort overall, and in those who withdrew within the first year of the study compared to those who did not.**

|  | **Overall** | **Completed Year 1** | **Withdrew in Year 1** | **p ***** |
| --- | --- | --- | --- | --- |
| # of mothers | 138** | 113 (81.9) | 25 (18.1) |  |
| # of competed surveys* | 42 (27-45) | 43 (37-46) | 12 (5-21) | <0.001 (a) |
| **SURVEY TYPE: percent of responses** | | | | |
| By IVR * | 63.0 (0-95.7) | 61.5 (0-95.5) | 66.7 (0-100) | 0.41 (a) |
| By email* | 0 (0-100) | 0 (0-100) | 0 (0-0) | 0.06 (a) |
| By person-to-person * | 0 (0-4.9) | 0 (0-4.5) | 0 (0-18.2) | 0.43 (a) |
| By other methods* | 2.1 (0-10.8) | 2.1 (0-7) | 0 (0-33.3) | 0.30 (a) |
| **DEMOGRAPHIC CHARACTERISTICS** | | | | |
| Age (mean, SD) | 30.8 (5.9) | 31.1 (5.6) | 29.4 (7.0) | 0.20 (c) |
| Years of education (mean, SD) | 13.3 (4.2) | 13.6 (4.3) | 12 (3.9) | 0.09 (c) |
| Ethnicity: Hispanic | 85 (61.6) | 70 (82.4) | 43 (81.1) | 0.86 (b) |
| Preferred language: Spanish | 48 (34.8) | 40 (35.4) | 8 (32.0) | 0.75 (a) |
| US-born | 54 (39.1) | 44 (38.9) | 10 (40) | 0.55 (b) |
| Crowding* | 1.2 (0.8-1.8) | 1.1 (0.8-1.8) | 1.3 (0.8-2.0) | 0.55 (a) |
| Minors in the house* | 1 (1-2) | 1 (1-2) | 1 (0-2) | 0.12 (a) |

* Median (Q1-Q3).

** This analysis included mothers who responded to a single survey (we have typically considered only those who completed at least two surveys).

*** P value compares mothers who remained vs. those who withdrew: (a) Wilcoxon signed rank test; (b) Chi square test; (c) t test. (Note that under “Survey Type”, the individual comparisons examine the non-parametric distributions of percent of responses by each method alone in those who withdrew and those who did not.)

IVR: interactive voice response, SD: standard deviation.
